# The Interplay of Genes and Environment Across Multiple Studies (IGEMS) Consortium After Fifteen Years

**DOI:** 10.1017/thg.2025.10036

**Published:** 2025-12-19

**Authors:** Deborah Finkel, Brian K. Finch, Margaret Gatz, Kaare Christensen, Carol E. Franz, Ida K. Karlsson, William S. Kremen, Robert F. Krueger, Michelle Lupton, Nicholas Martin, Matt McGue, Miriam A. Mosing, Jenae Neiderhiser, Marianne Nygaard, Elizabeth Prom-Worley, Chandra Reynolds, Perminder Sachdev, Elina Sillanpää, Eero Vuoksimaa, Keith E. Whitfield, Orla Hayden, Ellen Walters, Nancy L. Pedersen

**Affiliations:** 1 Center for Economic and Social Research, University of Southern California, Los Angeles, California, USA; 2 Department of Psychology, University of Southern California, Los Angeles, California, USA; 3 Institute for Gerontology, Jönköping University, Jönköping, Sweden; 4 Department of Sociology & Spatial Sciences, University of Southern California, Los Angeles, California, USA; 5 The Danish Twin Registry and Danish Aging Research Center, Department of Public Health, University of Southern Denmark, Odense, Denmark; 6 Department of Clinical Genetics, Odense University Hospital, Odense, Denmark; 7 Department of Biochemistry, Odense University Hospital, Denmark; 8 Department of Psychiatry, University of California, San Diego, La Jolla, California, USA; 9 Center for Behavior Genetics of Aging, University of California, San Diego, La Jolla, California, USA; 10 Department of Medical Epidemiology and Biostatistics, Karolinska Institutet, Stockholm, Sweden; 11 Department of Psychology, University of Minnesota, Minneapolis, Minnesota, USA; 12 QIMR Berghofer Medical Research Institute, Brisbane, Queensland, Australia; 13 Department of Neuroscience, Karolinska Institutet, Stockholm, Sweden; 14 Department of Cognitive Neuropsychology, Max Planck Institute for Empirical Aesthetics, Frankfurt am Main, Germany; 15 Melbourne School of Psychological Sciences, Faculty of Medicine, Dentistry, and Health Sciences, University of Melbourne, Melbourne, Victoria, Australia; 16 Department of Psychology, The Pennsylvania State University, University Park, Pennsylvania, USA; 17 Epidemiology, Biostatistics and Biodemography, Department of Public Health, University of Southern Denmark, Odense, Denmark; 18 Department of Epidemiology, Virginia Commonwealth University, Richmond, Virginia, USA; 19 Department of Psychology and Neuroscience, University of Colorado Boulder, Colorado, USA; 20 Department of Psychology, University of California Riverside, California, USA; 21 Centre for Healthy Brain Ageing (CHeBA), School of Clinical Medicine, UNSW Sydney, Sydney, New South Wales, Australia; 22 Faculty of Sport and Health Sciences, University of Jyväskylä, Jyväskylä, Finland; 23 Wellbeing Services County of Central Finland, Jyväskylä, Finland; 24 Institute for Molecular Medicine Finland FIMM, University of Helsinki, Helsinki, Finland; 25 Johns Hopkins Center for Health Disparities Solutions, Johns Hopkins University School of Public Health, Baltimore, Maryland, USA

**Keywords:** Aging, Dementia, Early life adversity, Gene–environment interplay, Health, Socioeconomic status

## Abstract

The Interplay of Genes and Environment across Multiple Studies (IGEMS) is a consortium of 21 twin studies from 5 countries (Australia, Denmark, Finland, Sweden, and United States) established to explore the nature of gene–environment interplay in cognitive, physical, and emotional health across the adult lifespan. The combined data from over 145,000 participants (aged 18 to 108 years at intake) has supported multiple research projects over the three phases of development since its inception in 2010. Phases 1 and 2 focused on launching and growing the consortium and supported important developments in data harmonization, analyses of data pooled across multiple studies, incorporation of linkages to national registries and conscription data, and integration of molecular genetic and classical twin designs. IGEMS Phase 3 focuses on developing appropriate infrastructure to maximize utilization of this large twin consortium for aging research.

The Interplay of Genes and Environment across Multiple Studies (IGEMS) consortium is both a data resource and a fellowship of interdisciplinary researchers (N. L. Pedersen et al., 2013; N. L. Pedersen et al., 2019). The purpose of the IGEMS consortium is to understand how prospective measures of risk factors measured earlier in the lifespan are associated with diverse outcomes including physical functioning (health, functional ability), and psychological functioning (wellbeing, cognition), particularly in later life. While twin studies are sometimes perceived as a specialized methodology, they are uniquely powerful for uncovering gene–environment interactions, gene–environment correlations, and controlling for major confounders such as family factors (both genetic and shared environmental) that help strengthen causal inferences where randomized controlled trials are not feasible. To apply the statistical designs/models to detect the mechanisms underlying such associations, extensive statistical power is needed, often more than a single study can provide.

IGEMS includes 145,011 twins from 21 studies representing 5 countries (Australia, Denmark, Finland, Sweden, and U.S.). The consortium includes primarily population-based studies with significant socioeconomic diversity that span a wide age range (18 to 108 years at intake) and has sufficient statistical power to address scientific questions that nontwin and smaller twin studies cannot. A set of well-characterized longitudinal phenotypes, including measures of physical health, cognitive health, and emotional health, and measures of multiple facets of adult socioeconomic status (SES) and rearing SES that are harmonized over time and across studies has been created. IGEMS also includes clinical diagnoses and linkage to diagnostic codes for Alzheimer’s disease and Alzheimer’s disease related dementia (AD/ADRD) where available. IGEMS has computed polygenic scores for 19,352 individuals in multiple domains, including education, Alzheimer’s disease, cognition, physical health, and emotional health.

As an international consortium with harmonized measures of risk and contextual factors assessed longitudinally on a large number of twins, IGEMS is particularly well suited for investigating the contribution of GE interplay to functioning in multiple domains across adulthood. In addition, IGEMS twins span multiple countries and a wide breadth of birth cohorts which allow for both: (a) the investigation of the impact of major policy changes on phenotypes and (b) the exploration of gene–environment interplay across multiple social and generational contexts.

## IGEMS Studies

From an original consortium of eight twin studies (Pedersen et al., 2013), IGEMS has expanded to include 21 studies from five countries, representing many of the most significant available longitudinal twin studies of adulthood and aging in the world. The total sample size is now 145,011, including members of 23,811 monozygotic (MZ) pairs and 41,063 dizygotic (DZ) pairs. The summary below outlines the sampling principles for each study. Numbers of pairs and age ranges at intake are provided in Table 1, as well as the number of waves and length of follow-up, where appropriate. Total *N*s refer to individuals and include members of incomplete pairs. Intake age indicates the age at which twins were first recruited into a project. For several studies, additional data are available from the originating registry prior to recruitment, or from registry linkages established before the study began. Instances of this are indicated in the descriptions below. Note that updates for several of the individual studies in IGEMS are separately included in this issue.

### Australia

The Australian Over 50’s study (A50) is based on a questionnaire mailed between 1993 and 1995 to Australian twins aged 50–95 recruited from the Australian Twin Registry (Mosing et al., 2009). The Older Australian Twins Study (OATS) were recruited beginning in 2006 from the Australian Twin Register and additional recruitment efforts. OATS incorporates in-person assessments every two years of twins aged 65 and older in the three eastern states of Australia: New South Wales, Victoria, and Queensland (Sachdev et al., 2009). For both A50 and OATS, earlier data from the Australian Twin Register are also available. The Prospective Imaging Study of Ageing: Genes, Brain and Behavior (PISA) is a prospective cohort study of midlife and older Australian adults with high and low genetic risk for dementia. The sample is drawn from an existing Australian GWAS cohort of twins and their family members. Only the twin sample is part of IGEMS. Data were collected from 2017–2022 and the twins ranged from 45 to 77 years of age (Lupton et al., 2021).

### Denmark

Danish studies are drawn from the Danish Twin Registry. The Longitudinal Study of Aging Danish Twins (LSADT) began in 1995 with the assessment of members of like-sex twin pairs born in Denmark prior to 1920 (McGue & Christensen, 2007). The Middle Age Danish Twins (MADT) study began in 1998 and includes twins ranging in age from 46 to 68 years at the original assessment (D. A. Pedersen et al., 2019). The MIddle age (MIDT) study carried out from 2008 to 2011 includes twins representing members of the Danish Twin Registry for the birth years 1931 through 1969 not already participating in MADT.

### Finland

The older Finnish Twin Cohort (FTC) study started 50 years ago; it was initiated by contacting all same-sex Finnish twin pairs born before 1958 with both co-twins alive in 1975 (Kaprio & Koskenvuo, 2002). Data collections for the cohort were conducted in 1975, 1981, 1990 and 2011, and several smaller substudies have included onsite visits and phone interviews (Kaprio et al., 2019).

### Sweden

Swedish studies are drawn from the population-based Swedish Twin Registry (STR). The Swedish Adoption/Twin Study of Aging (SATSA) began in 1984 (Finkel & Pedersen, 2004). The base population comprises all pairs of twins from the registry who indicated that they had been separated before the age of 11 and reared apart, and a sample of twins reared together matched on the basis of gender, date and county of birth. The OCTO-Twin Study (Origins of Variance in the Old-Old) included twin pairs who were over the age of 80 at baseline in 1991 (McClearn et al., 1997). Aging in Women and Men: A Longitudinal Study of Gender Differences in Health Behaviour and Health among Elderly (GENDER) is a study of opposite-sex twin pairs born between 1906 and 1925, with data collection begun in 1994 (Gold et al., 2002). The Twin and Offspring Study in Sweden (TOSS), begun in 1997, includes pairs of same-sex twins and their adolescent offspring (Neiderhiser & Lichtenstein, 2008). The Study of Dementia in Swedish Twins (HARMONY) was conducted between 1998 and 2004. Beginning in 1998, HARMONY screened all surviving twins from the STR age 65 and over in the Screening Across the Lifespan of Twins (SALT) effort (Lichtenstein et al., 2006) and clinically assessed those who screened positive or whose co-twin screened positive for cognitive impairment (Gatz et al., 2005). The Swedish studies in IGEMS can be linked to both questionnaires from 1967 or 1973 and administrative data including conscription and birth records for some birth years.

### United States

Each US study consists of an independent sample. The National Academy of Sciences-National Research Council (NAS-NRC) Twin Registry consists of white male twin pairs born in the years 1917 to 1927, both of whom served in the armed forces, mostly during World War II (Gatz, Harris et al., 2015). The intake questionnaire was in 1967–1973 when the men were aged 40–56. Information recorded at the time of the men’s induction into the military are available. The Mid-Atlantic Twin Registry (MATR) is a population-based registry established in 1975 of more than 60,000 twins primarily born or living in Virginia, North Carolina and South Carolina (Lilley et al., 2019). Multiple surveys have been conducted with the MATR twins. The Minnesota Twin Study of Adult Development and Aging (MTSADA) is a population-based sample drawn from state birth records (Finkel & McGue, 1993; McGue et al., 1993) and assessed beginning in 1986. Midlife in the United States (MIDUS) is a national telephone/mail survey originally carried out in 1995–1996 that included specific recruitment methods to accrue a sufficient sample of twins (South & Krueger, 2012). The Carolina African-American Twin Study of Aging (CAATSA) used public records to identify all living African-American twins in the state of North Carolina born between 1920 and 1970 (Whitfield, 2013); participants were recruited beginning in 1999. The Vietnam Era Twin Study of Aging (VETSA) is a community dwelling sample of male–male twin pairs, all of whom served in some branch of US military service sometime between 1965 and 1975 (Kremen et al., 2013). The first wave of VETSA data collection began in 2003. The Project Talent Twin Registry (PTTR) includes 2397 twins who responded to either the Project Talent Twin and Sibling Study (PTTS) in 2014 (ages 68–72) or the Project Talent Aging Study (PTAS) in 2019 (ages 73–77; Prescott et al., 2013). These samples were drawn from Project Talent (PT), a longitudinal study begun in 1960 with a nationally representative sample of U.S. high school students born 1942–1946 (Flanagan, 1962) from which data can be linked to twins in PTTR. The Colorado Adoption/Twin Study of Lifespan behavioral development and cognitive aging (CATSLife) is a prospective study of adult development with an assessment conducted between 2015 to 2021 at ages 28–51 years building on detailed early life assessments from the Colorado Adoption Study and Longitudinal Twin Study (Wadsworth et al., 2019).

## IGEMS Measures

Measures used in IGEMS analyses include aging-relevant outcomes in three broad domains: physical health and functional ability (e.g., self-reported diseases, subjective health, body mass index, grip strength, motor function, activities of daily living), psychological wellbeing (e.g., depressive symptoms, anxiety symptoms, subjective wellbeing, loneliness), and cognitive health (i.e., scores on cognitive tests; dementia). Predictors and covariates include health behaviors (e.g., smoking, alcohol, physical activity, cognitively engaging leisure activity), social resources, and indicators of SES. Table 2 presents a list of some of the primary phenotypes assessed and the number of IGEMS studies that include each variable.

Because participating studies differed in how similar constructs were assessed, IGEMS places emphasis on harmonizing relevant phenotypes and outcomes. Creating scores that are common across studies enables pooling data across samples, in order to increase statistical power. Score harmonization requires overlapping item content across studies as well as across time for longitudinal hypotheses. For some measures, it has been straightforward to create a common metric; for example, BMI, lung function, and blood pressure. For harmonizing education and occupation, we have recoded all studies to the International Standard Classification of Education (ISCED; UNESCO Institute for Statistics, 2012), and the International Standard Classification of Occupations (ISCO; Ganzeboom et al., 1992) as an international standard. Where a common metric was not already available, overlapping item content and response formats were identified and item response theory (IRT) or factor-analytic techniques were implemented to create harmonized scores across studies. When no common items were available across studies, IGEMS collected separate samples administered with the different scales used to measure a given construct, and used those results to establish ‘crosswalks’ between the scales (Gatz, Reynolds et al., 2015).

## Fifteen Years of IGEMS

**Figure 1.**
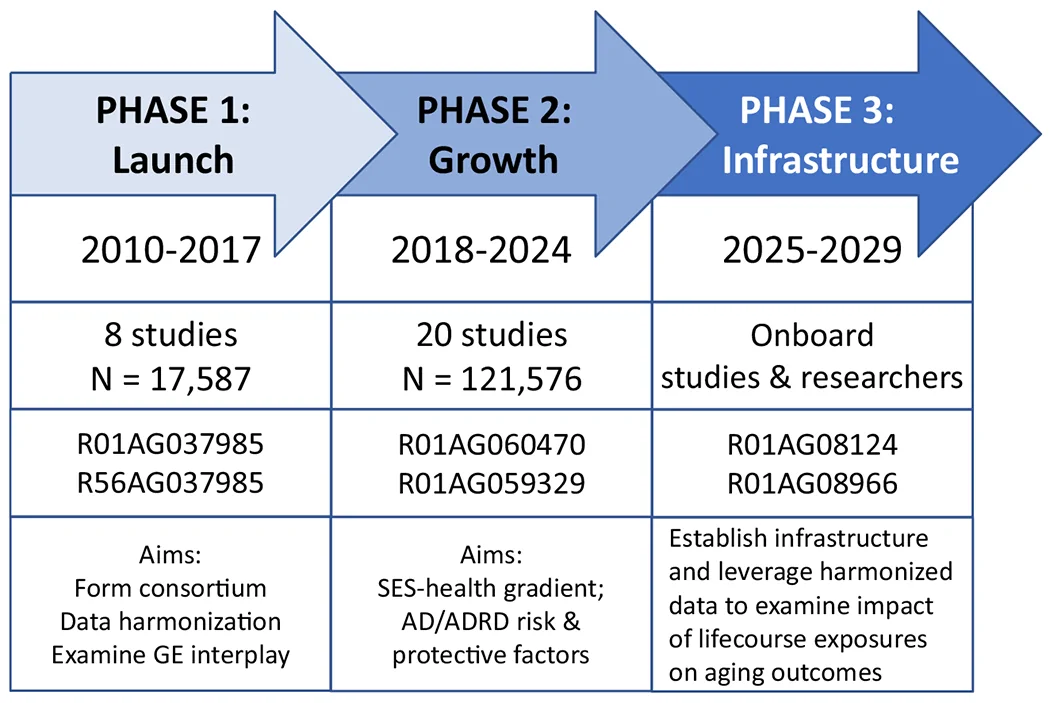
Phases of the IGEMS Consortium.

IGEMS has greatly expanded over the past 15 years (see Figure 1), and we continue to onboard new studies and grow research collaborations. Phase 1 of IGEMS, described by N. L. Pedersen et al. (2013) and proposed in response to an RFA and funded by R01 AG037985 (Pedersen), focused on establishing a collaboration among existing twin studies of aging to provide the rich phenotyping, genotyping, and high power necessary to investigate models of gene–environment interplay of social contexts and aging-related outcomes (N. L. Pedersen et al., 2013). IGEMS began with five twin cohorts and quickly grew to nine during this initial phase. Data harmonization protocols were tested and established (Gatz, Reynolds et al., 2015), and procedures for requesting and sharing data were instituted. Foundational analyses of harmonized variables were completed (Franz et al., 2013; Mosing et al., 2016; Pahlen et al., 2018) and multiple methods for leveraging twin data and genotyping to test models of GE-interplay were implemented (Finkel et al., 2016; Petersen et al., 2016; Petkus et al., 2017; Reynolds et al., 2016). For example, Reynolds and colleagues (2016) not only used a within-MZ-pair approach to investigate G × E interaction for body mass index, depressive symptoms, and cognitive measures, but they also incorporated the Alzheimer’s genetic marker APOE in the models as a potential source of the ‘G’ in G × E. They found that G × E interaction effects were evident in all three countries represented in the dataset. Moreover, they found evidence that APOE may represent a variability gene for depressive symptoms and spatial reasoning, because it was associated not just with trait mean but also with trait variability. In addition to launching the IGEMS consortium, Phase 1 resulted in 36 peer-reviewed publications.

Phase 2 of IGEMS focused on growth of the consortium and expansion of the scientific aims as described by Finch et al. (2019) and N. L. Pedersen et al. (2019). Twelve additional twin cohorts of aging joined IGEMS, adding one country (Australia) and increasing the total IGEMS sample by more than 700%. Although funding and scientific aims were divided into two projects, centralized data management and harmonization efforts, biweekly zoom meetings of the full IGEMS team, and annual in-person meetings continued to nurture the collaboration. In addition, we began using the powerful combination of twin methodology with molecular genetics in the form of polygenic scores. One project, led by Pedersen, Finch, and Gatz (R01 AG059329), focused on the frequently cited health ‘gradient’ for SES — the continuous, monotonic association between SES and health across the full spectrum of SES — which cannot be explained solely by poorer health among the most disadvantaged (Adler et al., 1994). IGEMS is unique in integrating individual- and country-level contributors to health gradients in twin studies to understand GE-interplay. Analysis results indicate moderation of genetic and environmental influences on health, cognition, frailty, and mortality by subjective and objective measures of SES (Ericsson et al., 2017; Finkel et al., 2022; Zavala et al., 2018). Sleep, loneliness, and smoking have been investigated as mechanisms linking the SES-health gradient to physical and cognitive health (Pahlen et al., 2021; Phillips et al., 2023; Vo et al., 2022), while country-level processes have also been explored (Finch, 2021; Finch et al., 2022; Gatz et al., 2018). The second Phase 2 project, led by Gatz and Pedersen (R01 AG060470), examined midlife and later-life risk and protective factors for AD/ADRD. In addition to harmonizing memory and verbal fluency measures that can indicate early cognitive decline (Gustavson et al., 2021; Luczak et al., 2023), the team derived and validated a latent dementia index using cognitive tests and measures of instrumental activities of daily living (Beam et al., 2022). Having this index allows inclusion of studies without clinical dementia diagnoses in analyses of risk and protective factors. Researchers investigated mechanisms of the relationship between BMI and AD/ADRD (Karlsson et al., 2020; Karlsson et al., 2021; Karlsson et al., 2022). Investigations of sex differences in AD/ADRD mechanisms (Beam et al., 2020; Karlsson et al., 2021; Luo et al., 2020) and the role of polygenic scores and DNA methylation (Karlsson et al., 2022; Karlsson et al., 2023; Reynolds et al., 2020) were also completed. A third project, led by Panizzon (R21 AG074212) focused on the physiological mechanisms of sex differences in ADRD. Identification of a U-shaped association between age at natural menopause and dementia risk challenged simplistic interpretations of the role of estrogen (Saelzler et al., 2024). To date, Phase 2 has produced over 100 peer-reviewed publications.

IGEMS infrastructure includes a data management team that conducts and documents data harmonization and maintains documentation of studies that are members of IGEMS, as well as a project manager to coordinate IGEMS researchers and projects. The primary goals of Phase 3 are to scale up the infrastructure of the consortium to support expansion of (a) the database by increasing the number of study cohorts, the number of harmonized variables, and the diversity of the IGEMS sample, and (b) the research team by inviting and supporting more researchers to join the IGEMS consortium. These developments will allow IGEMS to substantially extend the scientific focus beyond ADRD and to explore the effect of *lifecourse exposures* on health and functioning in multiple domains (Wagner et al., 2024), with particular interest in international comparisons of health disparities and differences in contexts that may drive *cross-national variations* in health outcomes (National Institutes of Health [NIH], 2024).

Interest in the effects of *life-course* exposures on the experience of aging has been growing over the past 20 years (Arcaya et al., 2016; Fratiglioni et al., 2004; Wagner et al., 2024), motivated by recognition that the genome alone cannot explain all of the variance in aging outcomes (Argentieri et al., 2023; Wild, 2005; Zhang et al., 2021). Studies of risk and protective factors stemming from the environment have made substantial contributions to our understanding of aging processes (Darin-Mattsson et al., 2017; Wolfova et al., 2021). One of the greatest challenges in epidemiology is drawing causal inferences from observational data. Although twin designs do not solve this problem, they strengthen the inferences that can be made by allowing the partition of risk into genetic, shared environment, and non-shared environment components (Duncan et al., 2014; McGue et al., 2010). Twin designs can also identify relationships driven by familial confounding, providing strong tests of causality.

Moreover, exposures will clearly *differ across age, cohorts, and countries*. Use of twin samples from multiple countries allows IGEMS to explore the impact of exposures and inequalities using different birth cohorts of twins compared at different ages within and between countries. This approach is important for several reasons, including: (a) heritability varies by social context (Uchiyama et al., 2022), and country-level differences in heritability are becoming more apparent (Branigan et al., 2013; Cesarini & Visscher, 2016; Min et al., 2013); (b) the exposure-outcomes relationships (e.g., education-ADRD) vary by context, making country and birth cohort differences suitable for comparing contexts (Beckfield et al., 2013; Chmielewski & Reardon, 2016; Mackenbach et al., 2008; Olafsdottir, 2007; Präg et al., 2016); (c) as implied by cross-national variation in heritability, G × E relationships may vary by country (Colodro-Conde et al., 2015) — for example, evidence suggests that gene by SES effects on intelligence vary widely by country with more egalitarian countries showing a reversal or zeroing out of these effects (Tucker-Drob & Bates, 2016); and (d) if exposures are based more on social class than on cognitive functioning or principles of equal access, then the exposure-outcome link may appear weakened (Sharp & Gatz, 2011), suggesting that the underlying cause may be genetically influenced functioning, not exposure per se.

All IGEMS cohorts include country and birth cohort identifiers, enabling the linking of macro-level economic data to examine direct effects as well as gene–environment and environment–environment interactions. For example, IGEMS has merged data from the Global Database on Intergenerational Mobility (GDIM, 2023), which provides country- and cohort-specific correlations in educational attainment between parents and offspring, and between mothers and daughters, by year and country. In addition, IGEMS has incorporated data from the World Inequality Database (van der Weide et al., 2024) —a publicly available resource that documents global income, education, and wealth disparities, including top income and wealth shares, measures of educational inequality, GINI coefficients, percentile distributions of income, and long-term trends in inequality both within and across countries. IGEMS researchers can specify the most relevant year of data based on hypothesized mechanisms; for example, income inequality at age 20 may be most pertinent to job market outcomes or income, whereas educational inequality at age 10 may be most relevant for educational attainment.

Recently funded IGEMS projects support the infrastructure and substantive goals of Phase 3. The aims of R01AG081248 (Finch/Finkel/Gatz) are to investigate mechanisms of educational influences on cognitive functioning and ADRD risk at multiple levels: genetic (polygenic score), individual, intergenerational (parental education), and environmental (GINI score for education), while investigating the impact of women’s differential access to educational and occupational opportunities across cohorts and countries. A recent expansion of the project focuses on increasing the number of African American twins in the IGEMS database to explore whether increasing access to education for women and African Americans influences dementia incidence as well whether the relative contribution of genetic influences may vary by gender or race/ethnicity. Responding directly to the Lancet report on modifiable risk factors for ADRD (Livingston et al., 2024; Livingston et al., 2020), R01AG089666 (Reynolds/Neiderhiser) will apply twin models to strengthen or refute causal hypotheses and test gene–environment interplay among modifiable factors for ADRD, considering risk and resilience within and across developmental periods of the lifespan. The project will evaluate the role of early-, mid-, and late-life physical health, health behaviors, and socioemotional factors, with a particular focus on how early life risk factors work together with mid- and late-life health and socioemotional factors to influence ADRD risk and resilience. This project will also begin to take advantage of sibling and adoption designs that are embedded in several of the twin studies now being onboarded as well as in CAATSA and PTTR.

Finally, R21AG087486 (Luczak) examines alcohol consumption as a modifiable risk factor for ADRD. This exploratory/developmental research study will have the power to model alcohol risk for ADRD in nuanced ways and broaden our understanding of how and why alcohol may affect ADRD risk. This project leverages 50 years of data to understand differences in these relationships between men and women and how they intersect with the *APOE* gene, the strongest genetic risk factor for ADRD. These studies comprising IGEMS Phase 3 will enhance understanding of the interplay between genetic and environmental factors in ADRD risk and protection and will identify potential mechanisms essential for developing effective interventions to reduce the burden of ADRD.

## Challenges

Building, maintaining, and growing an international consortium of twin cohorts is not an easy task, and the IGEMS team has faced and continues to face many challenges. Logistical challenges include coordination of data and researchers across multiple countries. Many of the twin cohorts in IGEMS are in EU countries and thus subject to General Data Protection Regulations (GDPR). As a result, although pooled analyses are the goal of most IGEMS projects, in some cases data-sharing protocols will only support parallel or meta-analyses. Careful development and implementation of data-use agreements are vital to support coordination across sites. Analytical challenges arise from the variety in birth cohorts, ages of intake, year of intake, length of follow-up, and sample sizes across studies. For example, multiple methods for data harmonization have been assessed and our efforts indicate that no one method is appropriate for all variables (Gatz, Reynolds et al., 2015). Differences in sample sizes and composition require exploration of weighting schemes or replication of results using ‘leave one out’ approaches to investigate the impact of any given twin cohort. Harmonizing PGS scores across studies with different genotyping platforms and methods requires careful coordination. Finally, issues that any twin study, longitudinal study, or study of aging may face (e.g., censoring, selective survival) can be multiplied in a consortium of multiple twin cohorts with different recruitment procedures, age cohorts, and follow-up periods.

## Summary

The IGEMS consortium harnesses a combination of twin designs and multiple studies representing different cohorts and contexts. The accomplishments of the consortium demonstrate the feasibility of this type of collaboration in addressing G × E interplay with respect to important age-related outcomes. In addition to increasing diversity through onboarding, we can increase the historical coverage of birth cohorts around the world and increase our sample sizes with more recent studies that include genotyping. Researchers interested in information about IGEMS variables, IGEMS harmonization methods, access to analytical scripts, proposing an analysis using IGEMS data, or joining the IGEMS research consortium are encouraged to visit https://dornsife.usc.edu/cesr/igems. In consultation with the IGEMS data management team, individual researchers can develop an abstract of intent describing a proposed project, which is presented to the IGEMS team for approval. Representatives of twin cohorts interested in joining IGEMS are encouraged to contact the IGEMS leadership team at IGEMS.Consortium@gmail.com.

## Figures and Tables

**Table 1.** Description of IGEMS study cohorts

Country	Study	MZ pairs	DZ pairs	Total *N*	Total # waves	Years span	Age range at intake	Earlier data available
Australia	A50	804	860	3622	2	20	50–95	Y
	OATS	160	125	599	4	7	65–-90	Y
	PISA	259	192	1709	5	41	45–77	Y
Denmark	LSADT	452	697	4731	6	10	70–102	Y
	MADT	670	1211	4308	2	11	46–68	Y
	MIDT	716	2200	10,231	1	NA	40–80	Y
Finland	FTC	3639	7721	31,145	4	36	18–-95	Y
Sweden	GENDER		647	1294	5	13	68–88	Y
	HARMONY	1200	3448	14,666	2	3	65–108	Y
	OCTO-Twin	150	201	702	5	11	79–97	Y
	SALT	4425	12240	44,994	1	5	41–103	Y
	SATSA	293	510	2210	17	30	26–95	Y
	TOSS	387	479	1745	1	NA	32–60	Y
United States	CAATSA	104	178	677	1	NA	22–-89	
	CATSLife	170	144	694	1	NA	28–36	Y
	MATR	3000	4364	11,650	1	8	18–89	
	MIDUS	345	533	1847	3	19	25–75	
	MTSADA	333	288	1359	3	11	25–92	
	NAS-NRC	7622	8790	20,398	9	39	40–84	Y
	PTTR	314	472	2397	2	7	67–77	Y
	VETSA	471	314	1608	3	16	51–71	Y
Total		23,811	41,063	145,011				

Note: MZ, monozygotic, DZ, dizygotic, OSDZ, opposite sex dizygotic pairs. Total *N* refers to individuals from both complete and incomplete pairs. Some individuals may have participated in more than one study; e.g., in A50 and OATS. The totals in the bottom row count each pair or individual once.

**Table 2.** Number of IGEMS studies with key variables

Variables	TOTAL N=21
**Socioeconomic status**	
International Standard Classification of Occupations	20
International Standard Classification of Education	21
Financial strain	14
Early life SES/Parental education	18
Marital status/Live alone	20
**Physical health**	
Measured blood pressure	14
Measured grip strength	12
Measured lung function	14
Weight, height, Body Mass Index	21
Self-reported diseases (cumulative illness)	21
Vascular risk (hypertension and diabetes)	20
Stroke and cardiovascular disease	20
Mortality	16
Measured motor function and balance	12
ADL/IADL	14
Subjective health	21
**Cognitive health**	
Cognitive test scores	17
Young adult cognitive ability	12
Mini-Mental State Examination or TICS	13
Clinical dementia diagnoses	9
Lagtent Dementia Index	11
**Emotional health**	
Anxiety (symptoms)	10
Depression (symptoms)	20
Subjective wellbeing	20
Loneliness	18
**Health behaviors**	
Cognitve complexity of occupation	20
Physical/Intellectual/social leisure activities	18
Alcohol	21
Smoking	21

## IGEMS consortium includes

Duke University: Brenda Plassman; Johns Hopkins University: Keith Whitfield; National Academies of Sciences, Engineering and Medicine: David Butler; University of Jyväskylä: Elina Sillanpää; Karolinska Institutet: Nancy Pedersen, Miriam Mosing, Malin Ericsson, Ida Karlsson; The Pennsylvania State University: Jenae Neiderhiser; QIMR Berghofer: Nicholas G. Martin, Michelle Lupton; University of California, San Diego: William Kremen, Carol Franz, Matthew Panizzon, Jeremy Elman; University of Colorado, Boulder: Chandra Reynolds, Daniel Gustavson; University of Helsinki: Eero Vuoksimaa; University of Minnesota: Matt McGue, Robert Krueger; University of New South Wales: Perminder Sachdev, Vibeke Catts, Teresa Lee, Karen Mather, Anbu Thalamuthu; University of Southern Denmark: Kaare Christensen, Marianne Nygaard; University of Southern California: Margaret Gatz, Brian Finch, Deborah Finkel, Christopher Beam, Susan Luczak, Thalida Em Arpawong, Andrew Petkus, Orla Hayden, Ellen Walters; Virginia Commonwealth University: Elizabeth Prom-Worley.
